# Supplementary material for: Integrative common and rare variant analyses provide insights into the genetic architecture of liver cirrhosis
Source: Nat Genet. 2024 Apr 17;56(5):827–37. doi: 10.1038/s41588-024-01720-y (PMC11096111; doi:10.1038/s41588-024-01720-y)
Supplement: Supplementary file 1 — Supplementary Note, Supplementary Figs. 1–6 and Consortia banners. [file 41588_2024_1720_MOESM1_ESM.pdf]

nature genetics

Article https://doi.org/10.1038/s41588-024-01720-y

Integrative common and rare variant analyses provide insights into the genetic architecture of liver cirrhosis

In the format provided by the authors and unedited

Table of Contents

SUPPLEMENTARY INFORMATION....2

Cohort information, case-control ascertainment, ethics and genotyping and imputation details 2

SUPPLEMENTARY FIGURES....8

Supplementary Figure 1. Effects of genetic variants on alcohol intake plotted against their effects on cirrhosis....8

Supplementary Figure 2. MR leave-one-out sensitivity analysis for the effect of alcohol (drinks per week) on cirrhosis....9

Supplementary Figure 3. Effects of genetic variants on BMI plotted against their effects on cirrhosis....10

Supplementary Figure 4. MR leave-one-out sensitivity analysis for the effect of BMI on cirrhosis 11

Supplementary Figure 5. Effects of genetic variants on IGF-1 plotted against their effects on cirrhosis....12

Supplementary Figure 6. MR leave-one-out sensitivity analysis for the effect of IGF-1 on cirrhosis....13

CONSORTIA BANNERS....14

REFERENCES....17

SUPPLEMENTARY INFORMATION

Cohort information, case-control ascertainment, ethics and genotyping and imputation details

Copenhagen Hospital Biobank Chronic Inflammatory Disease Cohort (CHB-CID) and Danish Blood Donor Study (DBDS)

Case-control ascertainment: The Copenhagen Hospital Biobank (CHB) Chronic Inflammatory Disease Cohort (CHB-CID) includes genome-wide genotype data for ~319,000 subjects admitted to general hospitals in the Copenhagen capital area of Denmark between 2009 and 2020.[1] The Danish Blood Donor Study is a cohort including blood donors from Denmark.[2] As of xxxx 2023, ~114,000 individuals with genome-wide genotype data were available. Cirrhosis cases were defined using the following ICD-10 codes: K70.3 ('alcoholic cirrhosis') or K74.6 ('other and unspecified cirrhosis') extracted from health records. The remaining individuals of the CHBCVDC/DBDS cohorts were included as controls. In total, 3,346 cirrhosis cases and 228,720 controls were identified. Cases with nonalcoholic fatty liver disease (NAFLD) were defined by ICD-10 code K76.0 (Fatty [change of] liver, not elsewhere classified), while cases with hepatocellular carcinoma (HCC) were defined by C22.0 (Liver cell carcinoma) or C22.9 (Malignant neoplasm of liver, not specified as primary or secondary).

Ethics: Since the biological samples stored in CHB- CVDC were based on leftover material from routine blood analyses, the patients were not asked for informed consent before inclusion. However, patients were informed about the opt-out possibility to have their biological specimens excluded from use in research in general. Thus, since 2004 a national Register on Tissue Application (Vævsanvendelsesregistret) lists all individuals who have chosen to opt out and whose samples cannot be used for research purposes. Before initiating this study, individuals listed in the Register on Tissue Application were excluded. For DBDS, informed consent was obtained from all participants. Both CHB- CVDC and DBDS are approved by the National Committee on Health Research Ethics (NVC 1708829 and NVC 1700407) and the Danish Data Protection Agency (P-2019-93 and P-2019-99).

Genotyping and imputation: Samples from 276,114 Danes from the CHB and DBDS were genotyped using Illumina Global Screening Array chips and long-range phased together with ~238,000 genotyped samples from North-western Europe using Eagle. Samples and variants with less than 98% yield were excluded. A haplotype reference panel was prepared in the same manner as for the Icelandic data (see below) by phasing whole-genome sequence genotypes of 15,576 individuals from Scandinavia, the Netherlands, and Ireland using the phased chip data. Graphtyper was used to call the genotypes which were subsequently imputed into the phased chip data. Whole genome sequencing, chip-typing, quality control, long-range phasing, and imputation from which the data for this analysis were generated was performed at deCODE genetics.

deCODE

Case-control ascertainment: Data from the deCODE study included 691 cirrhosis cases and 154,599 controls.[3] Cases were defined using the following ICD-10 codes: ICD10: K70.2 (Alcoholic fibrosis and sclerosis of liver), K70.3 (Alcoholic cirrhosis of liver), K70.4 (Alcoholic hepatic failure), K74.0 (Hepatic fibrosis), K74.1 (Hepatic sclerosis), K74.2 (Hepatic fibrosis with hepatic sclerosis), K74.6 (Other and unspecified cirrhosis of liver), K76.6 (Portal

hypertension), or I85 (Esophageal varices). The data was extracted from electronic health records from the Hospital Discharge Register at Landspitali ö the National University Hospital and Register of Primary Health Care Contacts, Register of Contacts with Medical Specialists in Private Practice and Causes of Death Register (stored at the Icelandic Directorate of Health). The cirrhosis cases were diagnosed during the period from 1970 until 2022.
Ethics: The study was approved by the National Bioethics Committee (VSN-16-036) and the Icelandic Data Protection Authority. Written informed consent was obtained from all participants who donated blood samples to research. All sample identifiers were encrypted in accordance with the regulations of the Icelandic Data Protection Authority.
Genotyping and imputation: The genome of the Icelandic population was characterized by whole-genome sequencing of 49,708 Icelanders using Illumina standard TruSeq methodology to a mean depth of 35x (SD 8x) with subsequent long-range phasing, (PMID 19165921) and imputing the information into 166,281 individuals chip-genotyped employing multiple Illumina platforms (PMID 25807286). Using genealogic information, we further imputed sequence variants into 285,664 relatives of the genotyped individuals to increase the sample sizes and power to detect associations. In total, we characterized up to 35.3 million variants in the Icelandic population with imputation score > 0.8.

Intermountain Healthcare
Case-control ascertainment: The HerediGene Population study is a large-scale collaboration between Intermountain Healthcare, deCODE genetics, and Amgen, Inc.[3] Participants in the HerediGene Population study are voluntary US residents over the age of 18 years, who gave permission to link anonymized genotypic data with EHRs. Cases were defined using the following ICD-10 codes: ICD10: K70.2 (Alcoholic fibrosis and sclerosis of liver), K70.3 (Alcoholic cirrhosis of liver), K70.4 (Alcoholic hepatic failure), K74.0 (Hepatic fibrosis), K74.1 (Hepatic sclerosis), K74.2 (Hepatic fibrosis with hepatic sclerosis), K74.6 (Other and unspecified cirrhosis of liver), K76.6 (Portal hypertension), or I85 (Esophageal varices). The remaining individuals of the Intermountain Healthcare cohort were included as controls. In total, 1,338 cases and 75,720 controls were identified.
Ethics: The Intermountain Healthcare Institutional Review Board approved this study, and all participants provided written informed consent prior to enrollment.
Genotyping and imputation: The Intermountain dataset was derived from a cohort created by whole-genome sequencing of 16,661 Americans of European ancestry living in Utah, to an average coverage of at least 20x, performed at deCODE genetics. These samples served as a reference panel for long-range phasing and imputation of 60,397 chip-typed individuals enrolled at multiple Intermountain Healthcare facilities.

UK Biobank
Case-control ascertainment: The UK Biobank (UKB) is a prospective cohort of more than 500,000 individuals living in the United Kingdom who were 40-79 years of age at recruitment (2006-2010).[4] Cases were defined using the following ICD-10 codes: ICD10: K70.2 (Alcoholic fibrosis and sclerosis of liver), K70.3 (Alcoholic cirrhosis of liver), K70.4 (Alcoholic hepatic failure), K71.7 (Toxic liver disease with fibrosis and cirrhosis of liver), K72.1 (Chronic hepatic failure), K74.0 (Hepatic fibrosis), K74.1 (Hepatic sclerosis), K74.2 (Hepatic fibrosis with hepatic sclerosis), or K74.6 (Other and unspecified cirrhosis of liver). All other participants were set as controls. Data from the UKB included 2,315 cirrhosis cases

and 423,701 controls. Cases with NAFLD were defined by ICD-10 code K76.0 (Fatty [change of] liver, not elsewhere classified), while cases with HCC were defined by C22.0 (Liver cell carcinoma) or C22.9 (Malignant neoplasm of liver, not specified as primary or secondary).
Ethics: The UK Biobank cohort has been approved by the Northwest Multicenter Research Ethics Committee, UK (Ref: 16/NW/0274). Written informed consent has been obtained from all study participants.
Genotyping and imputation: Genotypic data were available for 488,380 individuals and were imputed to the HRC, UK10K and 1,000 Genomes Phase 3 reference panels using IMPUTE4 to identify ≈ 93M variants for 487,409 individuals. Using the genotyped SNPs, persons were excluded if they had: high levels of missingness or heterozygosity, SNP genotype call rate < 98%, or if phenotypic and genotypic gender information was discordant.

Estonian Biobank
Case-control ascertainment: The Estonian Biobank is a volunteer-based sample of the Estonian adult population aged ≥18 years.[5] Baseline measurements included a standardized health examination, health-related questionnaires, blood samples for DNA, white blood cells and plasma tests, and clinical diagnoses defined by ICD-10 codes. Cirrhosis cases were defined using the following ICD-10 codes: K70.3 ('alcoholic cirrhosis') or K74.6 ('other and unspecified cirrhosis') extracted from health records.
Ethics: The Estonian Biobank has been approved by the Estonian Committee on Bioethics and Human Research, Estonian Ministry of Social Affairs. All participants provided written informed consent.
Genotyping and imputation: At present, more than 200,000 participants have undergone genotyping by a genome-wide SNP array which includes more than 700,000 SNPs (Illumina GSA microchip).

FinnGen Freeze 8
Case-control ascertainment: The summary statistics for 3,548 cases and 338,951 controls from Finngen[6] were downloaded from https://www.finngen.fi/en/access_results. Cases were defined using the following ICD-10 codes: ICD10: K70.2 (Alcoholic fibrosis and sclerosis of liver), K70.3 (Alcoholic cirrhosis of liver), K70.4 (Alcoholic hepatic failure), K74.0 (Hepatic fibrosis), K74.1 (Hepatic sclerosis), K74.2 (Hepatic fibrosis with hepatic sclerosis), K74.6 (Other and unspecified cirrhosis of liver), K76.6 (Portal hypertension), or I85 (Esophageal varices).
Ethics: Patients and control subjects in FinnGen provided informed consent for biobank research, based on the Finnish Biobank Act. Separate research cohorts, gathered before the Finnish Biobank Act was initialized (in September 2013) and start of FinnGen (August 2017), were collected based on study-specific consents and later assigned to the Finnish biobanks after approval by Fimea, the National Supervisory Authority for Welfare and Health. The Coordinating Ethics Committee of the Hospital District of Helsinki and Uusimaa (HUS) approved the FinnGen study protocol Nr HUS/990/2017. The FinnGen study is approved by Finnish Institute for Health and Welfare (THL), approval number THL/2031/6.02.00/2017, amendments THL/1101/5.05.00/2017, THL/341/6.02.00/2018, THL/2222/6.02.00/2018, THL/283/6.02.00/2019, THL/1721/5.05.00/2019, Digital and population data service agency VRK43431/2017-3, VRK/6909/2018-3, VRK/4415/2019-3 the Social Insurance Institution (KELA) KELA 58/522/2017, KELA 131/522/2018, KELA 70/522/2019, KELA 98/522/2019, and Statistics Finland TK-53-1041-17.7

Genotyping and imputation: A custom-made FinnGen ThermoFisher Axiom array (>650,000 SNPs) was used to genotype FinnGen samples. Genotype calls were made with AxiomGT1 algorithm. Individuals with ambiguous gender, high genotype missingness (>5%), excess heterozygosity (±4 SD), and non-Finnish ancestry were excluded. Variants with high missingness (>2%), low Hardy-Weinberg equilibrium (< 1×10^{-6}), or low minor allele count (<3) were excluded. High coverage (25–30×) WGS data were used to develop the Finnish population-specific SISu v3 imputation reference panel with Beagle 4.1. More than 16 million variants have been imputed (https://finngen.gitbook.io/documentation/methods/genotype-imputation).

Geisinger DiscovEHR
Case-control ascertainment: A total of 88,807 Geisinger Health System (GHS) patients who were enrolled in GHS's MyCode and Regeneron Genetics Center's DiscovEHR collaboration were included.[7] Cirrhosis cases were defined using the following ICD-10 codes: K70.3 (alcoholic cirrhosis) or K74.6 (other and unspecified cirrhosis) extracted from health records. A total of 1,939 cases and 86,868 controls were included.
Ethics: The GHS project has received ethical approval from the GHS Institutional Review Board. Participants have given informed consent to allow sharing of de-identified electronic health records, provide samples that can be linked to their health records for broad research, and permit re-contact for additional studies.
Genotyping and imputation: Genotyping was performed using the Illumina Human Omni Express Exome and Global Screening arrays. Imputation of untyped variants was performed using the TOPMed reference panel using the TOPMed imputation server.

AllofUS
Case-control ascertainment: A total of 1,258 cases with cirrhosis and 97,641 controls were included from the AllofUS cohort.[8] Cirrhosis cases were defined using SNOMED-19943007.
Ethics: Ethical approval was received from National Institute of Health All of Us Institutional Review Board. Participants provided written informed consent.
Genotyping and imputation: Genotyping was by whole genome sequencing (Illumina Whole Genome Sequencing. All of Us Research and Data Center whole genome sequencing Hail Matrix Table (v.6) was imported into Hail (v. 0.2.107). Samples were stratified by self-reported race or ethnicity into Black, Hispanic, and White subgroups. Samples were filtered for heterozygosity, sex mismatch, and genetic-ancestry outliers.

German/UK cirrhosis cohort
Case-control ascertainment: The summary statistics from a previously published GWAS[9] of 712 cases and 1465 controls (all self-reported German or UK ancestry) were downloaded from http://gengastro.med.tu-dresden.de/suppl/alc_cirrhosis/. Cases were defined as patients with clinically diagnosed or biopsy-proven cirrhosis on a background of past and/or present alcohol consumption of at least 60 g/day for women and 80 g/day for men for more than 10 years after exclusion of other causes of cirrhosis. Control individuals had no clinical or laboratory evidence of liver disease, confirmed by non-invasive assessment of liver fibrosis or examination of liver histology, on a background of alcohol dependence or reported alcohol consumption according to the criteria noted above.

Ethics: Patients gave written, informed consent and the study received approval from the ethics committees of all participating centres.
Genotyping and imputation: The German participants (n=410 cases and n=1,080 controls) were genotyped using the following four Illumina chips: OmniExpress array (Version 12v1_j), HumanHap550, Human610Quad and Human660wQuad BeadChip assays. The samples from the United Kingdom (n=302 cases and 346 controls) were genotyped using an Illumina OmniExpress chip (version 24v1-0_a). Pre-imputation quality control was done using PLINK (v.1.07). Individuals with genotyping success rate <97%, outlying autosomal heterozygosity, kinship coefficient <0.185, those failing gender check, and those that clustered outside the CEU HapMap population using multidimensional scaling were excluded. Only SNPs genotyped on all four arrays (N=298,405) were carried forward for imputation. Imputation was performed using IMPUTE 2 to reference 1000 Genomes Phase 3 (October 2014 release) or Phase 1 (March 2012 release). Post-imputation quality filtering was performed using a minimum impute info score of 0.8, HWE $>1\times10^{-6}$, and a minor allele frequency >1%, yielding 6,866,424 SNPs for the German and 7,871,013 SNPs for the UK data set. Quality control and phenotypic association analysis on this marker set was performed with SNPTEST (v2.5), using score statistic under an additive allelic effect model, including gender, age, BMI and type 2 diabetes status as covariates in the adjusted analyses. Study-specific effect estimates from the German and UK scans were genomic-control adjusted prior to a fixed-effect model meta-analysis using an inverse variance weighted method implemented in META 22. Meta-analysis was restricted to markers present in both data sets (N=6,770,425). The λ value was 1.005 for the combined meta-analysis.

Biobank Japan
Case-control ascertainment: The summary statistics for 2,551 cases and 176,175 controls from Biobank Japan[10] were downloaded from https://pheweb.jp/pheno/Cirrhosis. Cases were defined using ICD-10 code K74.6 (Other and unspecified cirrhosis of liver) or phecode 571.51, as described in Sakaue et al.[11]
Ethics: Biobank Japan received ethical approval from Research ethics committees at the Institute of Medical Science, the University of Tokyo, the RIKEN Yokohama Institute, and the 12 cooperating hospitals. All participants provided written informed consent.
Genotyping and imputation: Genotyping was done using the Illumina HumanOmniExpressExome BeadChip or a combination of the Illumina HumanOmniExpress and HumanExome BeadChips. Quality control (QC) of samples included exclusion of those with call rate < 0.98 and outliers from East Asian clusters identified by principal component analysis. For QC of genotypes, variants meeting any of the following criteria were excluded: (i) call rate < 99%, (ii) P value for HWE $< 1.0\times10^{-6}$, and (iii) number of heterozygotes less than five. Using 939 samples whose genotypes were also analyzed by whole genome sequencing (WGS), additional QC was done based on the concordance rate between genotyping array and WGS. Variants with a concordance rate < 99.5% or a non-reference discordance rate ≥ 0.5% were excluded. Imputation was by SHAPEIT (v2.778) and minimac3 (v2.0.1) and used data from 1000 Genomes Project Phase 3 (version 5) as a reference.

Million Veteran Program Cohort

Case-control ascertainment: A total of 21,689 cases with cirrhosis and 617,729 controls were included from the Million Veteran Program (MVP) Cohort.[12] Cirrhosis cases were defined using ICD-10 codes: K70.3 (Alcoholic cirrhosis of liver), K74.6 (Other and unspecified cirrhosis of liver), or ICD-9 codes 571.2 (Alcoholic cirrhosis of liver) or 571.5 (Cirrhosis without alcohol). Cases with NAFLD were defined using ICD-10 code K76.0.
Ethics: The MVP cohort was approved by VA Central Institutional Review Board. All participants provided written informed consent.
Genotyping and imputation: Genotyping was by a custom-made array (the 'MVP 1.0 array'), which is based on the Affymetrix Axiom Biobank Array and includes approximately 723,000 markers. Quality control procedures included a normalization procedure for mitigating plate-to-plate variation, cleaning and harmonizing genotype calls across batches, excluding samples with contamination, mislabeling, misidentification, >5% missingness of genotypes, or sex mismatch. Markers with >1% missingness as well as non-autosomal markers were removed. The QC procedures are described in detail in Hunter-Zink et al.[13]

SUPPLEMENTARY FIGURES

Supplementary Figure 1. Effects of genetic variants on alcohol intake plotted against their effects on cirrhosis. Alcohol intake variants were extracted from publicly available GWAS summary statistics in a European subpopulation (PMID: 36477530, N = 2,428,851) and cirrhosis (N_{case}=15,950) effects were from meta-analysis of 11 studies (i.e. without the UK Biobank). The x-axis depicts alcohol (drinks per week), in standard deviations. Points refer to effect estimates (logOR, measure of center) and error bars represent 95% CI. The lines depict the regression lines from four different MR methods: inverse variance weighted, MR Egger, and weighted median.

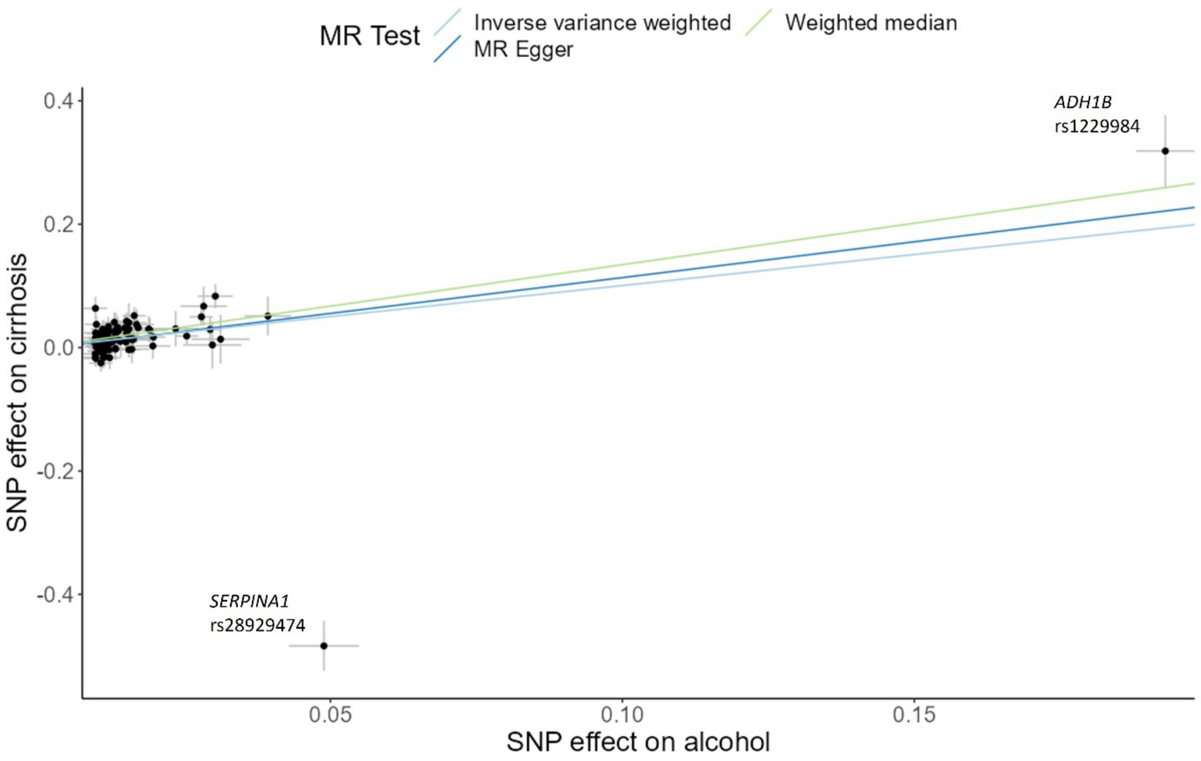

Supplementary Figure 2. MR leave-one-out sensitivity analysis for the effect of alcohol (drinks per week, N = 2,428,851) on cirrhosis (Ncase=15,950). For each genetic variant depicted on the Y-axis, the MR estimate has been recalculated without the specified variant. The MR-estimates (logOR, measure of center) and 95% confidence intervals (error bars) are from an inverse variance weighted method.

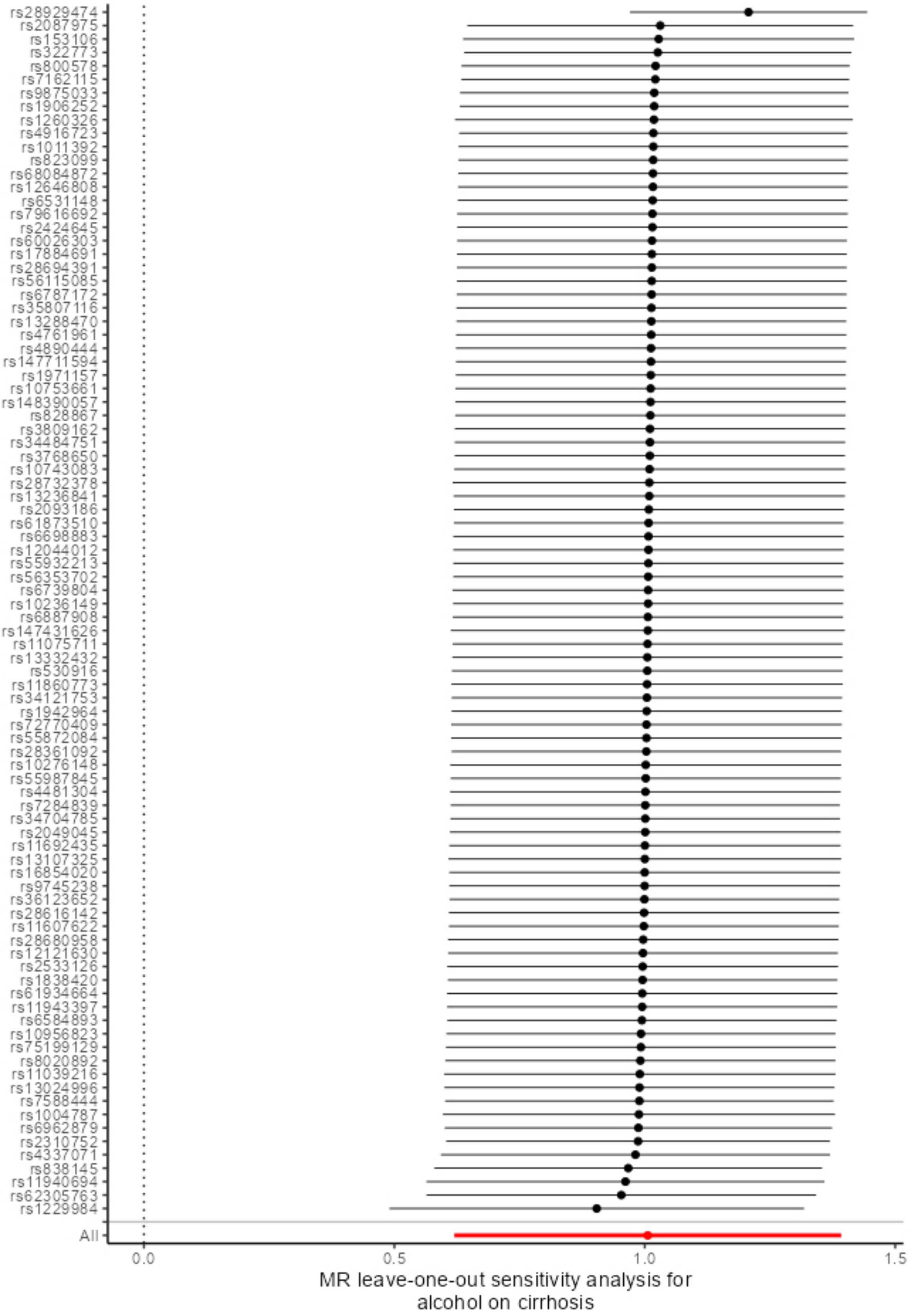

Supplementary Figure 3. Effects of genetic variants on BMI plotted against their effects on cirrhosis. Body mass index (N = 681.275) associated variants were extracted from MR-base (ieu-b-40) and cirrhosis (N_{case}=15,950) effects were from meta-analysis of 11 studies (i.e. without the UK Biobank). The x-axis depicts BMI (kg/m^2), in standard deviations. Points refer to effect estimates (logOR, measure of center) and error bars represent 95% CI. The lines depict the regression lines from four different MR methods: inverse variance weighted, MR Egger, and weighted median.

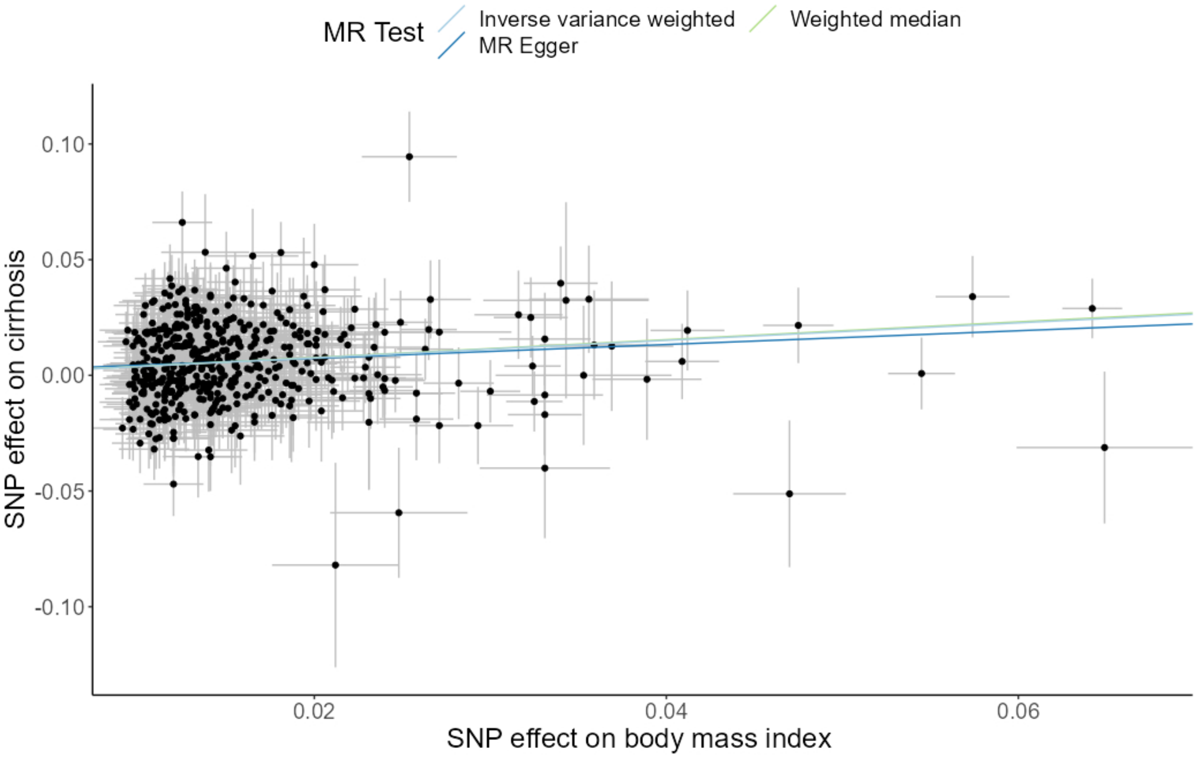

Supplementary Figure 4. MR leave-one-out sensitivity analysis for the effect of BMI (N = 681.275) on cirrhosis (Ncase=15,950). For each genetic variant depicted on the Y-axis, the MR-estimate has been recalculated without the specified variant. Due to the large number of BMI-associated genetic variants, individual rs-numbers are not included in the plot. The The MR-estimates (logOR, measure of center) and 95% confidence intervals (error bars) are from an inverse variance weighted method.

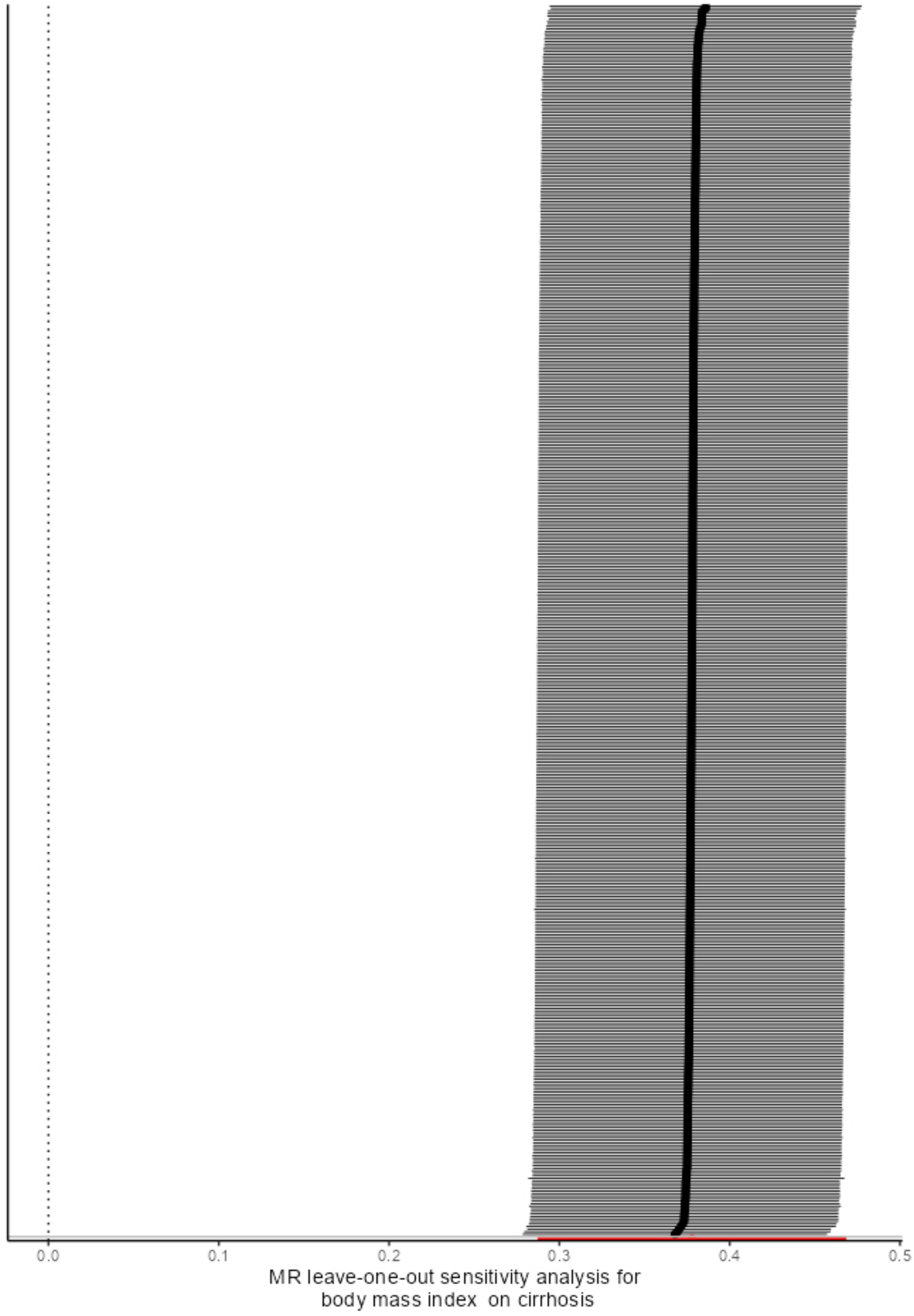

Supplementary Figure 5. Effects of genetic variants on IGF-1 plotted against their effects on cirrhosis. IGF-1 (N=342,439) associated variants were extracted from MR-base (ukb-d-30770_irnt) and cirrhosis (N_{case}=15,950) effects were from meta-analysis of 11 studies (i.e. without the UK Biobank). The x-axis depicts IGF-1, in standard deviations. Points refer to effect estimates (logOR, measure of center) and error bars represent 95% CI. The lines depict the regression lines from four different MR methods: inverse variance weighted, MR Egger, and weighted median.

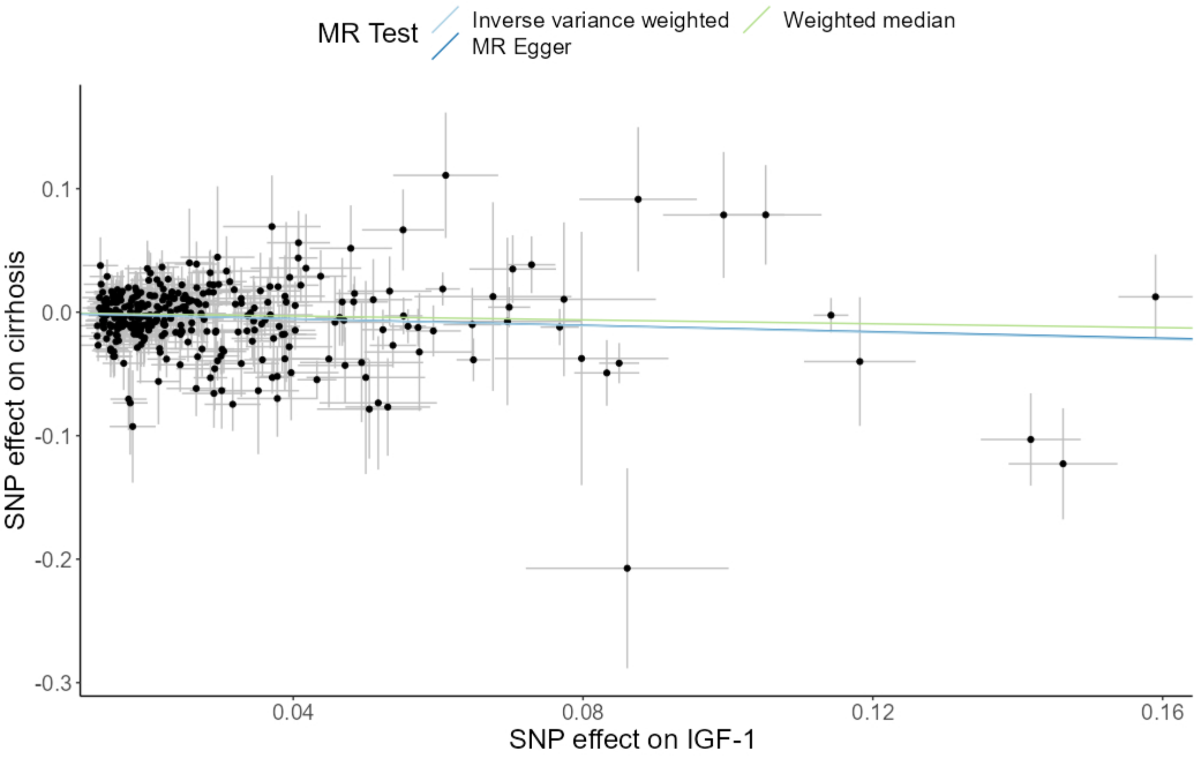

Supplementary Figure 6. MR leave-one-out sensitivity analysis for the effect of IGF-1 (N=342,439) on cirrhosis (Ncase=15,950). For each genetic variant depicted on the Y-axis, the MR-estimate has been recalculated without the specified variant. Due to the large number of IGF-1-associated genetic variants, individual rs-numbers are not included in the plot. The MR-estimates (logOR, measure of center) and 95% confidence intervals (error bars) are from an inverse variance weighted method.

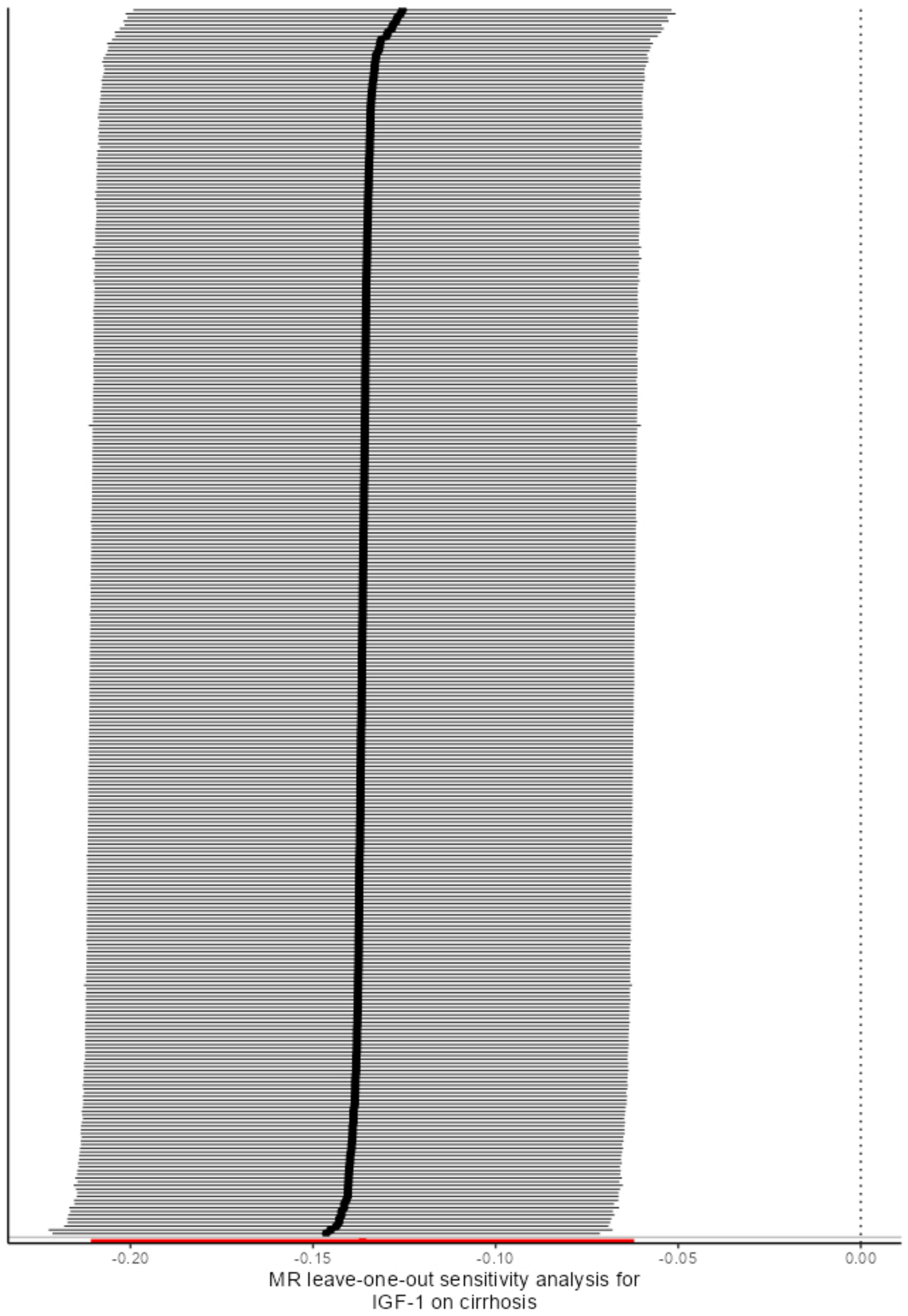

CONSORTIA BANNERS

Regeneron Genetics Center

Gonçalo Abecasis[1], Aris Baras[1], Aris Economides[1], Michael Cantor[1], Giovanni Coppola[1], Andrew Deubler[1], Aris Economides[1], Katia Karalis[1], Luca A Lotta[1], John D Overton[1], Jeffrey G. Reid[1], Katherine Siminovitch[1], Lyndon J Mitnaul[1], Alan Shuldiner[1], Christina Beechert[1], Caitlin Forsythe[1], Erin D Brian[1], Zhenhua Gu[1], Michael Lattari[1], Alexander Lopez[1], John D Overton[1], Maria Sotiropoulos Padilla[1], Manasi Pradhan[1], Kia Manoochehri[1], Ricardo Schiavo[1], Raymond Reynoso[1], Kristy Guevara[1], Laura M Cremona[1], Chenggu Wang[1], Hang Du[1], Sarah E Wolf[1], Amelia Averitt[1], Nilanjana Banerjee[1], Michael Cantor[1], Dadong Li[1], Sameer Malhotra[1], Deepika Sharma[1], Justin Mower[1], Jay Sundaram[1], Aaron Zhang[1], Sean Yu[1], Mudasar Sarwar[1], Jeffrey C Staples[1], Xiaodong Bai[1], Lance Zhang[1], Sean O'Keeffe[1], Andrew Bunyea[1], Lukas Habegger[1], Suganthi Balasubramanian[1], Suying Bao[1], Boris Boutkov[1], Gisu Eom[1], Lukas Habegger[1], Alicia Hawes[1], Olga Krasheninina[1], Rouel Lanche[1], Adam J Mansfield[1], Evan Edelstein[1], Sujit Gokhale[1], Alexander Gorovits[1], Evan K Maxwell[1], Ju Guan[1], George Mitra[1], Janice Clauer[1], Mona Nafde[1], Vrushali Mahajan[1], Razvan Panea[1], Koteswararao Makkena[1], Krishna Pawan Punuru[1], Benjamin Sultan[1], Sanjay Sreeram[1], Tommy Polanco[1], Ayesha Rasool[1], Jeffrey G. Reid[1], William J Salerno[1], Kathie Sun[1], Joshua Backman[1], Anthony Marcketta[1], Bin Ye[1], Lauren Gurski[1], Nan Lin[1], Gonçalo Abecasis[1], Jonathan Marchini[1], Manuel Allen Revez Ferreira[1], Yuxin Zou[1], Jack Kosmicki[1], Jonathan Ross[1], Joelle Mbatchou[1], Andrey Ziyatdinov[1], Kyoko Watanabe[1], Eli Stahl[1], Akropravo Ghosh[1], Lei Chen[1], Rujin Wang[1], Adam Locke[1], Carlo Sidore[1], Arden Moscati[1], Lee Dobbyn[1], Eric Jorgenson[1], Blair Zhang[1], Christopher Gillies[1], Michael Kessler[1], Maria Suciu[1], Timothy Thornton[1], Priyanka Nakka[1], Sheila Gaynor[1], Tyler Joseph[1] , Benjamin Geraghty[1], Anita Pandit[1], Joseph Herman[1], Sam Choi[1], Peter VandeHaar[1], Liron Ganel[1], Kuan-Han Wu[1], Aditeya Pandey[1], Kathy Burch[1], Adrian Campos[1], Scott Vrieze[1], Sailaja Vedantam[1], Charles Paulding[1], Amy Damask[1], Ariane Ayer[1], Aysegul Guvenek[1], George Hindy[1], Giovanni Coppola[1], Jan Freudenberg[1], Jonas Bovijn[1], Katherine Siminovitch[1], Luca A Lotta[1], Manav Kapoor[1], Mary Haas[1], Moeen Riaz[1], Niek Verweij[1], Olukayode Sosina[1], Parsa Akbari[1], Priyanka Nakka[1], Sahar Gelfman[1], Sujit Gokhale[1], Tanima De[1], Veera Rajagopal[1], Alan Shuldiner[1], Bin Ye[1], Gannie Tzoneva[1], Jin He[1], Adolfo Ferrando[1], Silvia Alvarez[1], Kayode Sosina[1], Neel Parikshak[1], Jacqueline Otto[1], Anna Alkelai[1], Vijay Kumar[1], Peter Dombos[1], Amit Joshi[1], Sarah Graham[1], Luanluan Sun[1], Antoine Baldassari[1], Jessie Brown[1], Cristen J Willer[1], Arthur Gilly[1], Hossein Khiabanian[1], Brian Hobbs[1], Billy Palmer[1], Juan Rodriguez-Flores[1], Esteban Chen[1], Jaimee Hernandez[1], Marcus B Jones[1], Michelle G. LeBlanc[1], Jason Mighty[1], Nirupama Nishtala[1], Nadia Rana[1], Jennifer Rico-Varela[1], Randi Schwartz[1], Thomas Coleman[1], Alison Fenney[1], Jody Hankins[1], Ruan Cox[1], Samuel Hart[1]

Geisinger-Regeneron DiscovEHR

Lance J. Adams[2], Jackie Blank[2], Adam Buchanan[2], David J. Carey[2], Kelly Cresci[2], F. Daniel Davis[2], Melissa Kelly[2], H. Lester Kirchner[2], Candice Laubach[2], Christa L. Martin[2], Cara Z. McCormick[2], Michelle Meyer[2], Tooraj Mirshahi[2], Matthew Oetjens[2], Kyle Retterer[2], Jules Savatt[2], Christopher Still[2], Marc Williams[2]

DBDS Genomic Consortium

Karina Banasik[3], Jakob Bay[4], Jens Kjærgaard Boldsen[5], Thorsten Brodersen[4], Søren Brunak[3], Kristoffer Burgdorf[3], Mona Ameri Chalmer[6], Maria Didriksen[7], Khoa Manh Dinh[5], Joseph Dowsett[7], Christian Erikstrup[5], Bjarke Feenstra[7], Frank Geller[7], Daniel Gudbjartsson[8], Thomas Folkmann Hansen[6], Lotte Hindhede[5], Henrik Hjalgrim[9], Rikke Louise Jacobsen[7], Gregor Jemec[10], Bitten Aagaard Jensen[11], Katrine Kaspersen[5], Bertram Dalskov Kjerulff[5], Lisette Kogelman[6], Margit Anita Hørup Larsen[7], Ioannis Louloudis[3], Agnete Lundgaard[3], Susan Mikkelsen[5], Christina Mikkelsen[7], Ioanna Nissen[7], Mette Nyegaard[12], Sisse Rye Ostrowski[7], Ole Birger Pedersen[4], Alexander Pil Henriksen[3], Palle Duun Rohde[12], Klaus Rostgaard[9], Michael Schwinn[7], Kari Stefansson[8], Hreinn Stefánsson[8], Erik Sørensen[7], Unnur Þorsteinsdóttir[8], Lise Wegner Thørner[7], Mie Topholm Bruun[13], Henrik Ullum[14], Thomas Werge[15], David Westergaard[3].

Estonian Biobank Research Team

Lili Milani[16], Andres Metspalu[16], Tõnu Esko[16], Mari Nelis[16], Georgi Hudjashov[16]

Million Veteran Program

Samuel M. Aguayo[17], Sunil K. Ahuja[18], Zuhair K. Ballas[19], Sujata Bhushan[20], Edward J. Boyko[21], David M. Cohen[22], John Concato[23], Joseph I. Constans[24], Louis J. Dellitalia[25], Joseph M. Fayad[26], Ronald S. Fernando[27], Hermes J. Florez[28], Melinda A. Gaddy[29], Saib S. Gappy[30], Gretchen Gibson[31], Michael Godschalk[32], Jennifer A. Greco[33], Samir Gupta[34], Salvador Gutierrez[35], Kimberly D. Hammer[36], Mark B. Hamner[37], John B. Harley[38], Adriana M. Hung[39], Mostaqul Huq40, Robin A. Hurley41, Pran R. Iruvanti42, Douglas J. Ivins43, Frank J.

Jacono44, Darshana N. Jhala45, Laurence S. Kaminsky46, Scott Kinlay47, Jon B. Klein48, Suthat Liangpunsakul49, Jack H. Lichy50, Stephen M. Mastorides51, Roy O. Mathew52, Kristin M. Mattocks53, Rachel McArdle54, Paul N. Meyer55, Laurence J. Meyer56, Jonathan P. Moorman57, Timothy R. Morgan[58], Maureen Murdoch[59], Xuan-Mai T. Nguyen47, Olaoluwa O. Okusaga[60], Kris-Ann K. Oursler[61], Nora R. Ratcliffe[62], Michael I. Rauchman[63], R. Brooks Robey[64], George W. Ross[65], Richard J. Servatius[66], Satish C. Sharma[67], Scott E. Sherman[68], Elif Sonel[69], Peruvemba Sriram[70], Todd Stapley[71], Robert T. Striker[72], Neeraj Tandon[73], Gerardo Villareal[74], Agnes S. Wallbom[75], John M. Wells[76], Jeffrey C. Whittle[77], Mary A. Whooley[78], Junzhe Xu[79], Shing-Shing Yeh[80], Michaela Aslan[23], Jessica V. Brewer[47], Mary T. Brophy[47], Todd Connor[82], Dean P. Argyres[81], Nhan V. Do[47], Elizabeth R. Hauser[83], Donald E. Humphries[47], Luis E. Selva[47], Shahpoor Shayan[47], Brady Stephens[84], Stacey B. Whitbourne[82], Hongyu Zhao[23], Jennifer Moser[50], Jean C. Beckham[82], Jim L. Breeling[47], JP Casas Romero[82], Grant D. Huang[50], Rachel B. Ramoni[47]

Affiliations:
[1] Regeneron Genetics Center, Tarrytown, NY, USA
[2] Geisinger Health System, Danville, PA, USA
[3] Novo Nordisk Foundation Center for Protein Research, Faculty of Health and Medical Sciences, University of Copenhagen, Copenhagen, Denmark
[4] Department of Clinical Immunology, Zealand University Hospital, Køge, Denmark
[5] Department of Clinical Immunology, Aarhus University Hospital, Aarhus, Denmark
[6] Danish Headache Center, Department of Neurology, Copenhagen University Hospital, Rigshospitalet-Glostrup, Copenhagen, Denmark
[7] Department of Clinical Immunology, Copenhagen University Hospital, Rigshospitalet, Copenhagen, Denmark
[8] deCODE Genetics, Reykjavik, Iceland
[9] Danish Cancer Society Research Center, Copenhagen, Denmark
[10] Department of Dermatology, Zealand University hospital, Roskilde, Denmark
[11] Department of Clinical Immunology, Aalborg University Hospital, Aalborg, Denmark
[12] Department of Health Science and Technology, Faculty of Medicine, Aalborg Univeristy, Aalborg, Denmark
[13] Department of Clinical Immunology, Odense University Hospital, Odense, Denmark
[14] Statens Serum Institut, Copenhagen, Denmark
[15] Institute of Biological Psychiatry, Mental Health Centre, Sct. Hans, Copenhagen University Hospital, Roskilde, Denmark
[16] Estonian Genome Centre, Institute of Genomics, University of Tartu, Tartu, Estonia
[17] Phoenix VA Health Care System, Phoenix, AZ USA
[18] South Texas Veterans Health Care System, San Antonio, TX USA
[19] Iowa City VA Health Care System, Iowa City, IA USA
[20] VA North Texas Health Care System, Dallas, TX USA
[21] VA Puget Sound Health Care System, Seattle, WA USA
[22] Portland VA Medical Center, Portland, OR USA
[23] VA Connecticut Healthcare System, West Haven, CT USA
[24] Southeast Louisiana Veterans Health Care System, New Orleans, LA USA
[25] Birmingham VA Medical Center, Birmingham, AL USA
[26] VA Southern Nevada Healthcare System, North Las Vegas, NV USA
[27] VA Loma Linda Healthcare System, Loma Linda, CA USA
[28] Miami VA Health Care System, Miami, FL USA
[29] VA Eastern Kansas Health Care System, Leavenworth, KS USA
[30] John D. Dingell VA Medical Center, Detroit, MI USA
[31] Fayetteville VA Medical Center, Fayetteville, AR USA
[32] Richmond VA Medical Center, Richmond, VA USA
[33] Sioux Falls VA Health Care System, Sioux Falls, SD USA
[34] VA San Diego Healthcare System, San Diego, CA USA
[35] Edward Hines Jr. VA Medical Center, Hines, IL USA
[36] Fargo VA Health Care System, Fargo, ND USA
[37] Ralph H. Johnson VA Medical Center, Charleston, SC USA
[38] Cincinnati VA Medical Center, Cincinnati, OH USA
[39] VA Tennessee Valley Healthcare System, Nashville, TN USA
[40] VA Sierra Nevada Health Care System, Reno, NV USA

[41] W.G. (Bill) Hefner VA Medical Center, Salisbury, NC USA
[42] Hampton VA Medical Center, Hampton, VA USA
[43] Eastern Oklahoma VA Health Care System, Muskogee, OK USA
[44] VA Northeast Ohio Healthcare System, Cleveland, OH USA
[45] Philadelphia VA Medical Center, Philadelphia, PA USA
[46] VA Health Care Upstate New York, Albany, NY USA
[47] VA Boston Healthcare System, Boston, MA USA
[48]Louisville VA Medical Center, Louisville, KY USA
[49] Richard Roudebush VA Medical Center, Indianapolis, IN USA
[50] Washington DC VA Medical Center, Washington, DC USA
[51] James A. Haley Veterans Hospital, Tampa, FL USA
[52] Columbia VA Health Care System, Columbia, SC USA
[53] Central Western Massachusetts Healthcare System, Leeds, MA USA
[54] Bay Pines VA Healthcare System, Bay Pines, FL USA
[55] Southern Arizona VA Health Care System, Tucson, AZ USA
[56] VA Salt Lake City Health Care System, Salt Lake City, UT USA
[57] James H. Quillen VA Medical Center, Johnson City, TN USA
[58] VA Long Beach Healthcare System, Long Beach, CA USA
[59] Minneapolis VA Health Care System, Minneapolis, MN USA
[60] Michael E. DeBakey VA Medical Center, Houston, TX USA
[61] Salem VA Medical Center, Salem, VA USA
[62] Manchester VA Medical Center, Manchester, NH USA
[63] St. Louis VA Health Care System, St. Louis, MO USA
[64] White River Junction VA Medical Center, White River Junction, VT USA
[65] VA Pacific Islands Health Care System, Honolulu, HI USA
[66] Syracuse VA Medical Center, Syracuse, NY USA
[67] Providence VA Medical Center, Providence, RI USA
[68] VA New York Harbor Healthcare System, New York, NY USA
[69] VA Pittsburgh Health Care System, Pittsburgh, PA USA
[70] North Florida/South Georgia Veterans Health System, Gainesville, FL USA
[71] VA Maine Healthcare System, Augusta, ME USA
[72] William S. Middleton Memorial Veterans Hospital, Madison, WI USA
[73] Overton Brooks VA Medical Center, Shreveport, LA USA
[74] New Mexico VA Health Care System, Albuquerque, NM USA
[75] VA Greater Los Angeles Health Care System, Los Angeles, CA USA
[76] Edith Nourse Rogers Memorial VA Hospital, Bedford, MA USA
[77] Clement J. Zablocki VA Medical Center, Milwaukee, WI USA
[78] San Francisco VA Health Care System, San Francisco, CA USA
[79] Atlanta VA Medical Center, Decatur, GA USA
[80] VA Western New York Healthcare System, Buffalo, NY USA
[81] Northport VA Medical Center, Northport, NY USA
[82] Raymond G. Murphy VA Medical Center, Albuquerque, NM USA
[83] Durham VA Medical Center, Durham, NC USA
[84] Canandaigua VA Medical Center, Canandaigua, NY USA

REFERENCES

1. Sørensen, E. *et al.* Data Resource Profile: The Copenhagen Hospital Biobank (CHB). *International Journal of Epidemiology* vol. 50 Preprint at https://doi.org/10.1093/ije/dyaa157 (2021).
2. Hansen, T. F. *et al.* DBDS Genomic Cohort, a prospective and comprehensive resource for integrative and temporal analysis of genetic, environmental and lifestyle factors affecting health of blood donors. *BMJ Open* **9**, (2019).
3. Sveinbjornsson, G. *et al.* Multiomics study of nonalcoholic fatty liver disease. *Nat Genet* **54**, (2022).
4. Sudlow, C. *et al.* UK Biobank: An Open Access Resource for Identifying the Causes of a Wide Range of Complex Diseases of Middle and Old Age. *PLoS Med* **12**, (2015).
5. Leitsalu, L. *et al.* Cohort profile: Estonian biobank of the Estonian genome center, university of Tartu. *Int J Epidemiol* **44**, (2015).
6. Kurki, M. I. *et al.* FinnGen provides genetic insights from a well-phenotyped isolated population. *Nature* **613**, (2023).
7. Akbari, P. *et al.* Sequencing of 640,000 exomes identifies GPR75 variants associated with protection from obesity. *Science (1979)* **373**, (2021).
8. The All of Us Research Program. *Nurse Pract* **43**, (2018).
9. Buch, S. *et al.* A genome-wide association study confirms PNPLA3 and identifies TM6SF2 and MBOAT7 as risk loci for alcohol-related cirrhosis. *Nat Genet* **47**, (2015).
10. Nagai, A. *et al.* Overview of the BioBank Japan Project: Study design and profile. *J Epidemiol* **27**, (2017).
11. Sakaue, S. *et al.* A cross-population atlas of genetic associations for 220 human phenotypes. *Nat Genet* **53**, (2021).
12. Gaziano, J. M. *et al.* Million Veteran Program: A mega-biobank to study genetic influences on health and disease. *J Clin Epidemiol* **70**, (2016).
13. Hunter-Zinck, H. *et al.* Genotyping Array Design and Data Quality Control in the Million Veteran Program. *Am J Hum Genet* **106**, (2020).
