# Supplementary material for: Integrative common and rare variant analyses provide insights into the genetic architecture of liver cirrhosis
Source: Nat Genet. 2024 Apr 17;56(5):827–37. doi: 10.1038/s41588-024-01720-y (PMC11096111; doi:10.1038/s41588-024-01720-y)
Supplement: Supplementary file 2 — Reporting Summary [file 41588_2024_1720_MOESM2_ESM.pdf]

nature portfolio

Corresponding author(s): Jonas Ghouse, Stefan Stender

Last updated by author(s): Mar 5, 2024

Reporting Summary

Nature Portfolio wishes to improve the reproducibility of the work that we publish. This form provides structure for consistency and transparency in reporting. For further information on Nature Portfolio policies, see our Editorial Policies and the Editorial Policy Checklist.

Statistics

For all statistical analyses, confirm that the following items are present in the figure legend, table legend, main text, or Methods section.

n/a	Confirmed	
☐	☒	The exact sample size (*n*) for each experimental group/condition, given as a discrete number and unit of measurement
☒	☐	A statement on whether measurements were taken from distinct samples or whether the same sample was measured repeatedly
☐	☒	The statistical test(s) used AND whether they are one- or two-sided<br>*Only common tests should be described solely by name; describe more complex techniques in the Methods section.*
☐	☒	A description of all covariates tested
☐	☒	A description of any assumptions or corrections, such as tests of normality and adjustment for multiple comparisons
☐	☒	A full description of the statistical parameters including central tendency (e.g. means) or other basic estimates (e.g. regression coefficient) AND variation (e.g. standard deviation) or associated estimates of uncertainty (e.g. confidence intervals)
☐	☒	For null hypothesis testing, the test statistic (e.g. *F*, *t*, *r*) with confidence intervals, effect sizes, degrees of freedom and *P* value noted<br>*Give P values as exact values whenever suitable.*
☒	☐	For Bayesian analysis, information on the choice of priors and Markov chain Monte Carlo settings
☒	☐	For hierarchical and complex designs, identification of the appropriate level for tests and full reporting of outcomes
☐	☒	Estimates of effect sizes (e.g. Cohen's *d*, Pearson's *r*), indicating how they were calculated

Our web collection on statistics for biologists contains articles on many of the points above.

Software and code

Policy information about availability of computer code

Data collection	No software was used for data collection.
Data analysis	The following software and packages were used for data analysis: PLINK 2.0 (https://www.cog-genomics.org/plink/2.0/), METAL v.2011-03-25 (http://csg.sph.umich.edu/abecasis/Metal/download/), MAGMA v.1.07 (https://ctg.cncr.nl/software/magma), EasyQC v.9.2 (https://www.uni-regensburg.de/medizin/epidemiologie-praeventivmedizin/genetische-epidemiologie/software/), LD score regression v.1.0.1 (https://github.com/bulik/ldsc), PoPS v.0.1 (https://github.com/FinucaneLab/pops/tree/add-license-1), PRS-CS v.2021-06-04 (https://github.com/getian107/PRScs/), REGENIE v.2.0.1 (https://rgcgithub.github.io/regenie/), TwoSampleMR v.0.5.6 (https://mrcieu.github.io/TwoSampleMR/), MVMR 0.4 (https://github.com/WSpiller/MVMR), pROC 1.18.4 (https://cran.r-project.org/web/packages/pROC/index.html) and Rv.4.1.2 (https://www.r-project.org/).

For manuscripts utilizing custom algorithms or software that are central to the research but not yet described in published literature, software must be made available to editors and reviewers. We strongly encourage code deposition in a community repository (e.g. GitHub). See the Nature Portfolio guidelines for submitting code & software for further information.

Data

Policy information about availability of data

All manuscripts must include a data availability statement. This statement should provide the following information, where applicable:

- Accession codes, unique identifiers, or web links for publicly available datasets
- A description of any restrictions on data availability
- For clinical datasets or third party data, please ensure that the statement adheres to our policy

GWAS meta-analysis summary statistics are available at GWAS Catalogue (GCST90319877 and GCST90319878). The cirrhosis PRS are available at PGS Catalog (PGS004621). Individual-level data sharing is subject to restrictions imposed by patient consent and local ethics review boards. The GTEx v.8 eQTL data used in this study are available in the GTEx Portal (https://gtexportal.org/home/datasets).

Human research participants

Policy information about studies involving human research participants and Sex and Gender in Research.

Reporting on sex and gender	We used biological sex throughout.
Population characteristics	Cohort characteristics (age and sex distribution) are provided in Supplementary Table 1 and each cohort has been described in more detail in Supplementary Information.
Recruitment	Recruitment information is provided in Supplementary Information.
Ethics oversight	All human research was approved within each contributing study by the relevant institutional review board and conducted according to the Declaration of Helsinki. CHB-CID/DBDS: National Committee on Health Research Ethics; deCODE: National Bioethics Committee; Intermountain Healthcare: Intermountain Healthcare Institutional Review Board; UKB: Northwest Multicenter Research Ethics Committee; Geisinger DiscovEHR: The GHS project has received ethical approval from the Geisinger Health System Institutional Review Board under project no. 2006-0258; FinnGen: The Coordinating Ethics Committee of the Hospital District of Helsinki and Uusimaa; Estonian Biobank: Ethical approval 1.1-12/624 from the Estonian Committee on Bioethics and Human Research, Estonian Ministry of Social Affairs; Biobank Japan: Research ethics committees at the Institute of Medical Science, the University of Tokyo, the RIKEN Yokohama Institute, and the 12 cooperating hospitals; Copenhagen General Population Study and Copenhagen City Heart Study: institutional review boards and Danish ethical committees; All of Us: National Institute of Health All of Us Institutional Review Board; and Dallas Liver Cohort: University of Texas Southwestern Institutional Review Board. MVP: VA Central Institutional Review Board (IRB). All participants (except for CHB-CID) provided written informed consent. For CHB-CID, patients were informed about the opt-out possibility of having their biological specimens excluded from use in research in general. Since 2004, a national Register on Tissue Application (Vævsanvendelsesregistret) lists all individuals who have chosen to opt out and whose samples cannot be used for research purposes. Before initiating this study, individuals listed in the Register on Tissue Application were excluded.

Note that full information on the approval of the study protocol must also be provided in the manuscript.

Field-specific reporting

Please select the one below that is the best fit for your research. If you are not sure, read the appropriate sections before making your selection.

☒ Life sciences ☐ Behavioural & social sciences ☐ Ecological, evolutionary & environmental sciences

For a reference copy of the document with all sections, see nature.com/documents/nr-reporting-summary-flat.pdf

Life sciences study design

All studies must disclose on these points even when the disclosure is negative.

Sample size	All available samples passing QC were used to maximize power.
Data exclusions	Within each contributing study, samples were excluded on the basis of well-established individual and variant quality control procedures to remove poor quality genotypes, SNPs and samples. Quality control filters are provided in Supplementary Table 1 and in under Methods.
Replication	We replicated significant variants once in the MVP cohort
Randomization	Randomization is not relevant since this is a retrospective case-control study and there was no treatment to randomize.
Blinding	Blinding was not relevant because it is a retrospective case-control study and there was no randomized allocation to be blinded to.

Reporting for specific materials, systems and methods

We require information from authors about some types of materials, experimental systems and methods used in many studies. Here, indicate whether each material, system or method listed is relevant to your study. If you are not sure if a list item applies to your research, read the appropriate section before selecting a response.

Materials & experimental systems

n/a	Involved in the study
☒	☐ Antibodies
☒	☐ Eukaryotic cell lines
☒	☐ Palaeontology and archaeology
☒	☐ Animals and other organisms
☒	☐ Clinical data
☒	☐ Dual use research of concern

Methods

n/a	Involved in the study
☒	☐ ChIP-seq
☒	☐ Flow cytometry
☒	☐ MRI-based neuroimaging
